# Development of a Web-Based Oxygenation Dashboard for Preterm Neonates: A Quality Improvement Initiative

**DOI:** 10.1007/s10916-024-02064-0

**Published:** 2024-04-24

**Authors:** J. A. Poppe, R. S. Smorenburg, T. G. Goos, H. R. Taal, I. K. M. Reiss, S. H. P. Simons

**Affiliations:** 1 Department of Neonatal and Paediatric Intensive Care, Division of Neonatology, Erasmus MC Sophia Children’s Hospital, University Medical Center Rotterdam, Rotterdam, The Netherlands; 2 Department of Biomechanical Engineering, Delft University of Technology, Delft, The Netherlands

**Keywords:** Prematurity, Neonatal intensive care unit, Oxygenation monitoring, Dashboard

## Abstract

Background: Preterm neonates are extensively monitored to require strict oxygen target attainment for optimal outcomes. In daily practice, detailed oxygenation data are hardly used and crucial patterns may be missed due to the snapshot presentations and subjective observations. This study aimed to develop a web-based dashboard with both detailed and summarized oxygenation data in real-time and to test its feasibility to support clinical decision making. Methods: Data from pulse oximeters and ventilators were synchronized and stored to enable real-time and retrospective trend visualizations in a web-based viewer. The dashboard was designed based on interviews with clinicians. A preliminary version was evaluated during daily clinical rounds. The routine evaluation of the respiratory condition of neonates (gestational age < 32 weeks) with respiratory support at the NICU was compared to an assessment with the assistance of the dashboard. Results: The web-based dashboard included data on the oxygen saturation (SpO_2_), fraction of inspired oxygen (FiO_2_), SpO_2_/FiO_2_ ratio, and area < 80% and > 95% SpO_2_ curve during time intervals that could be varied. The distribution of SpO_2_ values was visualized as histograms. In 65% of the patient evaluations (*n* = 86) the level of hypoxia was assessed differently with the use of the dashboard. In 75% of the patients the dashboard was judged to provide added value for the clinicians in supporting clinical decisions. Conclusions: A web-based customized oxygenation dashboard for preterm neonates at the NICU was developed and found feasible during evaluation. More clear and objective information was found supportive for clinicians during the daily rounds in tailoring treatment strategies.

**Supplementary Information:**

The online version contains supplementary material available at 10.1007/s10916-024-02064-0.

## Introduction

The oxygenation of preterm neonates is monitored extensively at the neonatal intensive care unit (NICU). Oxygen saturations need to be managed within tight levels and necessitate careful titration of inspired oxygen to limit the risks of hyperoxia and hypoxia for retinopathy of prematurity and negative effects on brain development in the preterm infant, respectively [1–4]. In practice, maintaining the predefined oxygen saturation levels is challenging with a high proportion of time spent outside the target range and the occurrence of prolonged hypoxic and hyperoxic episodes [5–7]. The physiological monitor data are often not optimally used to guide respiration and oxygenation, with most of the data presented with a lack of detail and in snapshots at the bedside. Crucial signs and patterns in physiological data can easily be missed causing delay in clinician’s responsiveness or misinterpretations with a potential impact on the safety and outcomes of preterm neonates [8, 9].

Visualization of oxygenation trend and summary data could improve detection, interpretation, understanding, and evaluation in preterm infants, providing insight into potential needs for adaptations in respiratory support, pharmacotherapy and oxygen supply. This was observed in our earlier study with the example of pharmacotherapy, where the indication to start doxapram, a respiratory stimulant, and the evaluation of the clinical effect could potentially be improved using physiological trend data [10]. Also, the use of SpO_2_ histograms as summary measure, in some NICUs standard provided by bedside monitoring software, can increase the time spent within the SpO_2_ target limits [11, 12]. In addition, early changes in vital signs can be a useful indication of the adverse cardiorespiratory effects of analgesics in individuals [13]. In the past years, some clinical dashboards have already been developed in the medical setting for varying indications and users [14], including a decision support tool for morphine in neonates [15]. No third party software monitoring is yet available that fit the needs for the current clinical aims.

The aim of this study was to develop a web-based visualization dashboard with physiological trend data reflecting the oxygenation status of preterm infants to support real-time clinical decision making. In addition, the impact of the dashboard on the estimated level of hypoxia and respiratory instability was investigated and its feasibility to support clinical decision making was evaluated.

## Methods

### Study Design

The first steps of a quality improvement project were performed at the NICU of the Erasmus MC Sophia Children’s Hospital, the Netherlands. All data of preterm infants from bedside monitors (M540, Draegerwerk AG & Co KGaA, Lübeck, Germany), including SpO_2_, heart rate, and respiratory rate, and data from ventilation machines (ACUTRONIC Medical Systems AG, Hirzel, Switzerland) including FiO_2_, were automatically logged and stored, and were available for direct retrospective use to design a prototype of a web-based oxygenation viewer. The medical ethics review board of the Erasmus University Medical Center granted a waiver from approval for this study according to the Dutch Medical Research Involving Human Subjects Act (MEC-2018–1106).

### Data Acquisition and Prototype Development

**Fig. 1:**
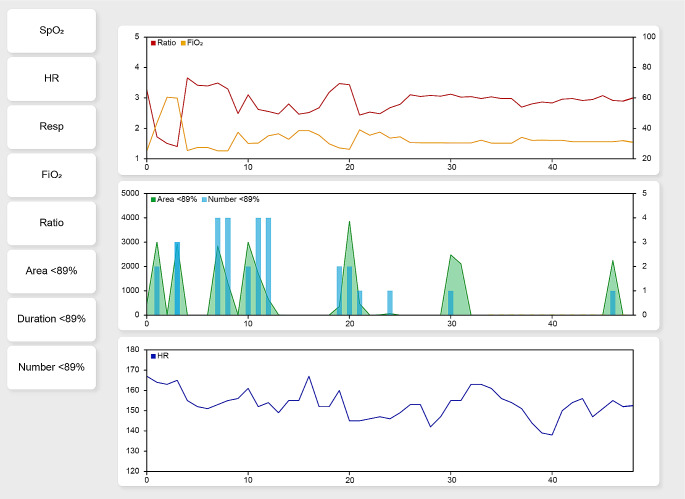
The platform that was used to support the interviews with clinicians to explore the most effective design of the prototype. Different parameters could be selected and inserted in one of the 3 graphs. The platform allowed to insert 2 parameters in one graph in a format of choice (line, bars, or area under the curve). This figure presents an example of a completed design with the x-axis representing the time in hours, the left y-axis the value of the first inserted parameter and the right y-axis the value of the second inserted parameter using predefined ranges. This figure presents an example with the mean SpO_2_/FiO_2_ ratio and the mean FiO_2_ (top graph), the area under the 89% SpO_2_ curve and the number of episodes of desaturation (middle graph), and the mean heart rate (bottom graph). The averaging interval of the data was set to 1 h. SpO_2_: oxygen saturation; HR: heart rate; Resp: respiratory rate; FiO_2_: fraction of inspired oxygen; Ratio: SpO_2_/FiO_2_ ratio; Area < 89%: area under the 89% SpO_2_ curve; Duration < 89%: duration of time spent below the 89% SpO_2_ target limit; Number < 89%: Number of times spent below the 89% SpO_2_ target limit

The development of the dashboard prototype was started with two rounds of interviews with clinicians, including neonatologists and nurses, who were affiliated to the department. The bedside monitors at the time of the interviews presented real-time values of the heart rate, SpO_2_, and respiratory rate, in addition with retrospective values from a couple of minutes. Changes in FiO_2_ values from ventilation machines were loaded into the electronic patient charts. In the first round clinicians were asked open questions in a structured format about what data a dashboard should present to support their decision making, and in which timeframe and format. For the second round of interviews a platform was developed using C# (version 7.2, Microsoft, Silicon valley, US), JavaScript (version ES6, Mozilla Foundation, US), HTML (version 5, W3C and WHATWG) and CSS (version 3, CSS Founder Pvt Ltd, India) that allowed users to select parameters in different formats (lines, bars, area under the curve) to design their own optimal dashboard (Fig. 1). Anonymized data, including heart rate, SpO_2_, respiratory rate, and FiO_2_, that were logged and saved could be loaded retrospectively into the platform. Alternative parameters were derived from the SpO_2_ and FiO_2_, including the SpO_2_/FiO_2_ ratio, the area under the 89% and above the 95% SpO_2_ curve (the local lower and upper limit), and the number and duration of episodes with a SpO_2_ value < 89% and > 95%.

**Fig. 2:**
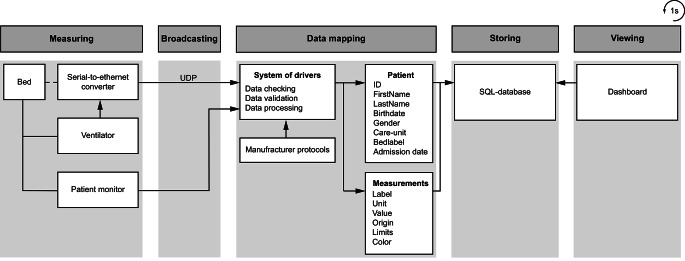
Schematic infrastructure for the process of data registration to real-time visualization. UDP = User Datagram Protocol; SQL = Structured Query Language

The input of the interviews formed the basis for the development of the prototype dashboard with a multi-disciplinary team of technicians, researchers and clinicians. The dashboards could only be viewed after authorization and double identification processes, in line with the hospital security rules. A schematic infrastructure of the data registration to real-time visualization is presented in Fig. 2. Data from the patient monitor were captured from the Dräger Infinity network and contained the measurements together with a bed-label and patient ID. The serial data measurements from the ventilators were converted into an Ethernet interface using a serial-to-ethernet converter (NPort 5610-16, Moxa, New Taipei City, Taiwan) that was connected to the medical devices. The serial-to-ethernet converter used the User Datagram Protocol (UDP) as a communication model to transform the serial data packages into UDP-packages. The packages consisted of ventilator settings and measurements, and mapped to a specific bed based on the UDP port the data was send to. A system consisting of drivers was developed in C# for data mapping, including data checking, validation, and processing, with the drivers programmed according to the protocol of each manufacturer of the medical device. The system was used to synchronize all data streams in time and to export these data into a beforehand developed relational SQL-database. Data were extracted from the database with the C# programming language using SQL Queries in the backend of the dashboard. The Asynchronous JavaScript And XML approach [16] was used to process the data in the frontend of the dashboard. The layout of the dashboard was formatted using HTML and CSS, and data were visualized using the open source library ApexCharts (MIT license).

Data of the patient monitor were stored once per second and every other second for the ventilators, the maximum available frequency at which they are available. The SpO_2_/FiO_2_ ratio was calculated by dividing the SpO_2_ by the FiO_2_ per second. The median with minimum and maximum values were calculated for the SpO_2_, SpO_2_/FiO_2_ ratio, and FiO_2_. The area < 80% SpO_2_ curve was calculated by multiplying the difference between the SpO_2_ limit of 80% and the measured SpO_2_ by the time spent below the SpO_2_ of 80% in a specified time averaging interval. The SpO_2_ limit of 80% was based on the study of Poets et al. [1] and selected to filter more severe desaturations instead of the less severe variations around the lower clinical limit in the support platform during the interviews. The area above the 95% SpO_2_ curve was calculated by multiplying the difference between the SpO_2_ limit of 95% and the measured SpO_2_ by the time spent above the SpO_2_ limit of 95% in a certain time interval. SpO_2_ values > 95% were not taken into account when FiO_2_ was ≤ 21%. The data were averaged in a 2 min interval to ensure data performance.

### Prototype Evaluation

The prototype of the oxygenation viewer was evaluated during daily clinical rounds between March and July 2021. The respiratory status of neonates (gestational age < 32 weeks) with respiratory support at the NICU was first assessed routinely, as was standard of clinical care, followed by a structured assessment with the assistance of the prototype dashboard during clinical rounds. Participants were asked to rate the level of hypoxia (scale 1 to 10, respectively from less hypoxia to more hypoxia), the degree of respiratory instability (scale 1 to 10, respectively from less stability to more stability), the source of information that was used during the evaluation without the prototype, suggested potential changes in therapy based on the evaluation with or without the prototype, and the potential added value of the prototype during a specific clinical round (Supplementary Information).

## Results

### Prototype Development

In the first round, 15 clinicians were interviewed, including 6 neonatologists and 9 nurses. Clinicians reported that vital parameters as presented at the current bedside monitor should be included in the prototype (*n* = 10), as well as incidents such as desaturations, bradycardia, and tachycardia (*n* = 9), and data on oxygen need (*n* = 3). The prototype was recommended to include trends and reference values for an individual patient. In the second round of interviews, clinicians (*n* = 5, including 3 nurses and 2 neonatologists) commented in more detail on the content and design of the prototype. SpO_2_, oxygen need, heart rate, and the relationship between incidents (including oxygen desaturations, apnea, and bradycardia) were reported mostly by the clinicians as required information.

**Fig. 3:**
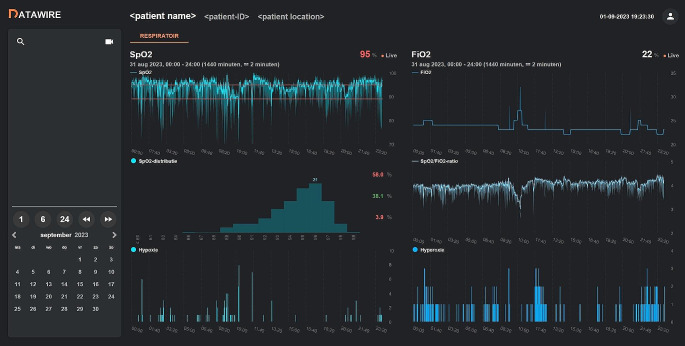
Prototype of the dashboard that was evaluated during clinical rounds. The prototype presents 24 h of anonymized data from a patient admitted to the neonatal intensive care unit, including data on the SpO_2_ (top graph left), SpO_2_ distribution (middle graph left), area < 80% SpO_2_ curve (defined as hypoxia - bottom graph left), FiO_2_ (top graph right), SpO_2_/FiO_2_ ratio (middle graph right), and area > 95% SpO_2_ curve (defined as hyperoxia - bottom graph right). The left column presents normally an overview of the admitted infants and a calendar to select a day and a certain time interval (1, 6, or 24 h). The figure presents that around 8:00 the median of the SpO_2_ decreased to below the clinical lower SpO_2_ limit of 89%, without an increase in FiO_2_ for almost 2 h. Other timeframes, especially before 3:00 and after 21:00, show median SpO_2_ levels above the upper SpO_2_ limit of 95% with a FiO_2_ that remains above 21%. Real-time availability of this information would possibly result in improved titration of the FiO_2_ to avoid SpO_2_ levels outside the clinical target limits, although this is a retrospective evaluation that could have been influence by other factors as well. SpO_2_: oxygen saturation; FiO_2_: fraction of inspired oxygen

The web-based prototype included data on the SpO_2_, FiO_2_, SpO_2_/FiO_2_ ratio, area < 80% SpO_2_ curve, area > 95% SpO_2_ curve, and SpO_2_ distribution during a variable (1, 6, or 24 h) time interval (Fig. 3). The development team decided to focus on SpO_2_ and FiO_2_ and the relation between these parameters to prevent an overload of information resulting in difficulties with interpretation of the visualized data. Data on heart rate and bradycardic events were, therefore, not included. Data on the SpO_2_, SpO_2_/FiO_2_ ratio, and FiO_2_ were presented as median with minimum and maximum values. The area < 80% SpO_2_ curve and the area > 95% SpO_2_ curve were presented in bars. The SpO_2_ data were also visualized as a histogram to present the distribution of SpO_2_ values in a certain period. The presented time interval could be easily set and changed to 1, 6–24 h according to the clinicians needs and wishes. The prototype could be accessed on any computer with a secured internet connection to the hospital.

### Prototype Evaluation

During daily clinical rounds, 86 evaluations were performed in 31 different patients by 11 assessors (10 neonatologists and 1 nurse specialist). In 4 cases the assessment with the prototype was not completed due to technical issues. The evaluation of the respiratory status without the prototype was mostly based on the report of the caregiving nurse (36%) and the electronic patient records (32%) (Table 1). The median score of both the respiratory instability and the level of hypoxia did not differ between the assessment with and without the prototype. However, the respiratory instability and level of hypoxia were scored equally in only resp. 59% and 35% of the cases when using the prototype compared to standard evaluation (Table 1).

In 81% of the cases the prototype provided improved information for the participating clinicians on the respiratory instability and/or level of hypoxia (74%) or other (7%), including the level of hyperoxia and a more continuous view on episodes of desaturation. In 74% of the patients the prototype was judged to have added value in supporting clinical decisions. In cases where the prototype did not have added value to the assessment, the respiratory stability and level of hypoxia were scored as 9 (8–9) and 2 (1–2) respectively, compared to 7 (7–8) and 3 (2–5) in the cases where the prototype did have added value.

**Table 1:** Findings of the evaluations during clinical rounds with and without the prototype

	Without prototype	With prototype
**Evaluations (** ***n*** **)**	86	82
**Assessors (** ***n*** **)** ^**a**^	11	11
**Patients (** ***n*** **)**	31	31
**Source of information**		
Electronic patient dossier	71 (32%)	
Report of caregiving nurse	81 (36%)	
Intercollegiate briefing	36 (16%)	
Bedside monitor	25 (11%)	
Other	11 (5%)	
**Respiratory instability** ^**b**^	8 (7–9)	
Median (IQR)		8 (7–9)
More instability (*n*)		15 (19%)
Equal instability (*n*)		48 (59%)
Less instability (*n*)		18 (22%)
**Level of hypoxia** ^**b**^	3 (2–5)	
Median (IQR)		3 (2–4)
More hypoxia (*n*)		31 (38%)
Equal hypoxia (*n*)		28 (35%)
Less hypoxia (*n*)		22 (27%)
**Better insight provided by the prototype**		
Yes, instability		12 (14%)
Yes, hypoxia		20 (24%)
Yes, both instability and hypoxia		30 (36%)
Yes, other reasons		6 (7%)
No		15 (18%)
**Added value to support policy making**		
No		20 (26%)
Yes, although same treatment policy		53 (68%)
Yes, changed to a different treatment policy		5 (6%)

^a^10 neonatologists, 1 nurse specialist

^b^Scale 1 to 10, respectively from less respiratory stability to more respiratory stability

^c^Scale 1 to 10, respectively from less hypoxia to more hypoxia

## Discussion

A web-based customized dashboard was developed presenting both detailed and summarized trend data on the oxygenation status of preterm neonates at the NICU. The dashboard was of added value to the participating clinicians in supporting clinical decision making, and the level of hypoxia and respiratory instability was estimated differently. However, the median estimated level of hypoxia and respiratory instability was comparable and it did not lead to alternative treatment strategies in most cases. The oxygenation status of preterm infants may fluctuate frequently, making adequate monitoring and treatment strategies challenging. As shown in this study, treatment decisions are mostly based on reports by the NICU staff, while it appears that nursing notes only account for 25–30% of true desaturation episodes, and neonatal medical notes around 7% [9]. Trend visualization of data on oxygenation could bring more awareness of potential hypoxia and hyperoxia and supports clinicians during daily clinical rounds in tailoring treatment strategies. This could lead to more timely and effective interventions or avoiding ineffective interventions.

Dashboards have the potential to transform a high volume of metrics from multiple data sources into real-time information through visual representations to impact accuracy, efficiency, user satisfaction, and quality and safety of care [8, 17]. A study by Strivatsa et al. showed downward trends in the rates of mortality, retinopathy of prematurity, and bronchopulmonary dysplasia after implementation of an Enhanced Pulse Oximetry Tool [18]. Their web-based tool provided real-time oxygen saturation monitoring, oxygen delivery monitoring, and analysis of cumulative patient data on the last 36 h. However, the study was performed over a 12 years’ time period and causality could not be assessed. Data visualization could improve situation awareness and adherence to clinical guidelines [8]. Recently, Middleton et al. showed that implementing behavioral change interventions, including automated oxygen saturation histogram printouts, resulted in significant improvement in targeted NICU staff behaviors related to oxygen saturation maintenance [19]. Further research is needed to investigate the added value of visualization dashboards next to currently used clinical alarms for timely interventions.

Improved ways to display physiological data trends may stimulate the clinician’s responsiveness in adding or weaning interventions based on the patient’s changing condition [20]. We already showed that physiological data can reflect the cardiorespiratory effect of red blood cell transfusions and the respiratory effect of doxapram that is used to treat apnea of prematurity, although the clinical impact of these findings need to be further investigated prospectively [10, 21]. Vinks et al. developed a model-informed and individualized pharmacokinetic profile aiming for enhanced individualized and evidence-based pharmacotherapy, resulting in better clinical efficacy and safety with fewer side effects [15]. Real-time visualization of physiological data provides the opportunity to guide decision making when cutoff values or objective rules are available. Fuerch et al. showed the impact of a decision support tool during neonatal resuscitation, resulting in significantly fewer deviations from protocol in a simulated setting [22]. Prediction algorithms on continuous physiological data could be used to provide early warning scores, as presented with the heart rate characteristic monitor using heart rate variability to predict sepsis [23]. However, providing more extensive or alternative information is not of added value in every case. The use of a respiratory function monitor, for example, did not lead to any change in ventilation of preterm infants at birth [24]. Clinical evaluation of newly developed tools is essential to avoid information overload without supporting cognitive reasoning.

A systematic review on emergency department quality dashboards concluded that dashboard development challenges include data sources and data quality, integration with other systems, adaptability of dashboard functionalities to user needs, and selection of performance indicators [17]. In this study, the added value of the dashboard according to the participating clinicians, the estimated level of hypoxia and hyperoxia, and the impact on clinical decision making were assessed as performance indicators. From the evaluation during clinical rounds, it appeared that in almost all cases the dashboard was of added value, and in the remaining cases the level of hypoxia was assessed to be minimal with a more stable respiratory condition. In 5 cases the dashboard would have influenced the treatment policy, according to the evaluation of the clinicians. In a majority of the cases the dashboard was judged to be of added value without influencing the treatment policy. This may result from a relatively stable respiratory condition in the evaluated infant, the lack of objective criteria for the currently displayed parameters and the lack of training of the participating clinicians. Sufficient training is essential before a prototype can be implemented into clinical care. Clinicians should be able to use the dashboard by themselves, and to interpret the presented information by each graph correctly. A learning curve is also expected over time. Reference values based on historical data from a comparable population and more objective scores, for example a histogram classification score [25, 26], could support data interpretation. SpO_2_ histograms were included in our prototype as these are not presented by the standard bedside monitoring software of the unit, in contrast to some other centers. Optimal thresholds to determine clinically significant respiratory events have not been established and future research is needed to investigate objective criteria for respiratory interventions, making a decision-guided dashboard possible.

The safety and quality of the presented data is also essential for correct interpretation and adequate decision making. The displayed levels of hypoxia and hyperoxia were extracted from data of pulse oximeters, while pulse oximetry data was found to not fulfil all the performance requirements for titrating oxygen supplementation in neonatal patients [27]. Clinicians should be aware of the data sources to interpret the displayed information correctly. All logged data need to be checked for measurement errors and artifacts as much as possible. Summary measures should be presented to dilute artifacts (by i.e. movement, rounds of daily care), although detailed information should remain available to detect deteriorations in the clinical condition. In this study, a two-minute averaging interval was selected to dilute artifacts while presenting information that is detailed enough to detect deteriorations.

Visualization tools have the potential to impact accuracy, efficiency, user satisfaction, quality or safety of care in the ICU, although many of the developed dashboards are solely evaluated with a qualitative approach and tested a single solution in a single implementation setting [8]. The currently developed dashboard also needs further investigation on the impact on patient outcomes and the generalisability to different populations. The dashboard is based on a specific data infrastructure and adjustment can be needed when applied to other units. In this study, the dashboard was mostly evaluated by neonatologists during clinical rounds, although nurses and, at some sites, respiratory therapists are a key user group. Further evaluations of the individual elements of the dashboard is needed to further simplify and improve the applicability of the dashboard. Future perspectives are to implement and evaluate the dashboard in a structured quality improvement project [28, 29]. Registration as a medical device would be required following the European Medical Device Regulation [30], to allow for decision making based on the presented data in the dashboard. The impact needs to be evaluated by measuring the level of hypoxia, oxygen need, fluctuations in the oxygen saturation and patient outcomes before, during and after implementation. This may guide clinicians in the future for timely interventions, clinical effect measurement, prevention of overtreatment and improved patient outcomes.

This study showed the first feasibility steps for a dashboard on oxygenation in preterm infants in the intensive care. The dashboard was of added value to support clinical decision making, by providing improved insight in the level of hypoxia and respiratory instability. Next steps would be to determine if the dashboard indeed improves neonatal treatment and outcome.

## Electronic Supplementary Material

Below is the link to the electronic supplementary material.

Supplementary Material 1

## Data Availability

No datasets were generated or analysed during the current study.
