# Supplementary material for: Development of a Web-Based Oxygenation Dashboard for Preterm Neonates: A Quality Improvement Initiative
Source: J Med Syst. 2024 Apr 24;48(1):46. doi: 10.1007/s10916-024-02064-0 (PMC11043117; doi:10.1007/s10916-024-02064-0)
Supplement: Supplementary file 1 — Supplementary Material 1 [file 10916_2024_2064_MOESM1_ESM.docx]

**DEVELOPMENT OF A WEB-BASED OXYGENATION DASHBOARD FOR PRETERM NEONATES: A QUALITY IMPROVEMENT INITIATIVE**

J.A. Poppe*^,1^, R.S. Smorenburg^1^, T.G. Goos^1,2^, H.R. Taal^1^, I.K.M. Reiss^1^, S.H.P. Simons^1^

**Affiliations**

1. Department of Neonatal and Paediatric Intensive Care, Division of Neonatology, Erasmus MC Sophia Children’s Hospital, University Medical Center Rotterdam, Rotterdam, The Netherlands
2. Department of Biomechanical Engineering, Delft University of Technology, Delft, The Netherlands.

* **Corresponding author**:

Jarinda A. Poppe, MSc, Department of Neonatal and Paediatric Intensive Care, Division of Neonatology, Erasmus MC Sophia Children’s Hospital, Doctor Molewaterplein 40, Rotterdam, 3015 GD, The Netherlands ([j.poppe@erasmusmc.nl](mailto:j.poppe@erasmusmc.nl)).

**EVALUATION FORM DURING DAILY CLINICAL ROUNDS**

**RESPIRATORY EVALUATION WITHOUT DASHBOARD**

| **Level of hypoxia** | | |
| --- | --- | --- |
| 1. | \| 1 \| 2 \| 3 \| 4 \| 5 \| 6 \| 7 \| 8 \| 9 \| 10 \| \| --- \| --- \| --- \| --- \| --- \| --- \| --- \| --- \| --- \| --- \| \| Unstable Completely stable \| \| \| \| \| \| \| \| \| \|   **Is the respiratory condition stabilized?** | |
| 2. | \| 1 \| 2 \| 3 \| 4 \| 5 \| 6 \| 7 \| 8 \| 9 \| 10 \| \| --- \| --- \| --- \| --- \| --- \| --- \| --- \| --- \| --- \| --- \| \| No hypoxia Too much hypoxia \| \| \| \| \| \| \| \| \| \|   **Does hypoxia exists and if yes, to what extend?** | |
| 3. | **This information is based on:**   - Registrations in EPD - Briefing - Nurse - Drager monitor - Other, namely………………………………………………………………………………. | |
| **Respiratory interventions** | | |
| 4. | **Was something changed in the respiratory treatment in the last 24 hours?**   - Yes - No | **If yes, what changed?**   - Ventilatory support - Caffeine - Doxapram - Other, namely………………………………….   ………………………………………………………… |
| 5. | **Would you change the support based on the current evaluation?**   - No, because …………………………………………………………………………………………………………..   ……………………………………………………………………………………………………………………………….   - Yes, decrease because ……………………………………………………………………………………………   ……………………………………………………………………………………………………………………………….   - Yes, increase because …………………………………………………………………………………………….   ………………………………………………………………………………………………………………………………. | |

**RESPIRATORY EVALUATION WITH DASHBOARD**

| **Level of hypoxia** | |
| --- | --- |
| 6. | **Is the respiratory condition stabilized?**   \| 1 \| 2 \| 3 \| 4 \| 5 \| 6 \| 7 \| 8 \| 9 \| 10 \| \| --- \| --- \| --- \| --- \| --- \| --- \| --- \| --- \| --- \| --- \| \| Unstable Completely stable \| \| \| \| \| \| \| \| \| \| |
| 7. | **Does hypoxia exists and if yes, to what extend?**   \| 1 \| 2 \| 3 \| 4 \| 5 \| 6 \| 7 \| 8 \| 9 \| 10 \| \| --- \| --- \| --- \| --- \| --- \| --- \| --- \| --- \| --- \| --- \| \| No hypoxia Too much hypoxia \| \| \| \| \| \| \| \| \| \| |
| 8. | **Does the dashboard provide additional insight in the respiratory condition and the level of hypoxia?**   - No - Yes, better insight in the stability - Yes, better insight in the level of hypoxia - Yes, better insight in both - Other……………………………………………………………………………………………………………………..   ………………………………………………………………………………………………………………………………. |
| **Respiratory interventions** | |
| 9. | **Does the dashboard provide support for estimation of the need/possibilities for changes in the respiratory support?**   - No - Yes, but the use of the dashboard did not result in adjusting the proposed policy - Yes, a different policy was chosen when using the dashboard namely……………………………………………………………………………………………………………………   ………………………………………………………………………………………………………………………………. |

**General remarks: ……………………………………………………………………………………………………………………………**

**………………………………………………………………………………………………………………………………………………….......**
